# Parasexuality in genitourinary investigations: a qualitative study

**DOI:** 10.1186/1756-0500-7-126

**Published:** 2014-03-06

**Authors:** Allyson Lipp, Chris Shaw, Paul Gill

**Affiliations:** 1 School of Care Sciences, Faculty of Life Sciences & Education, University of South Wales, Glyntaf, CF37 1DL Pontypridd, Wales, UK

**Keywords:** Male, Men, Masculinity, Patient, Genitourinary investigations, Gender, Sexuality, Parasexuality, Urology

## Abstract

**Background:**

Genitourinary investigations are performed on a large proportion of middle-aged and older men and the majority undergo investigations for prostate issues. The effects that genitourinary disease can have on men depend on the type of problem, investigations required and treatment including impotence, gynaecomastia and urinary incontinence that have lasting devastating physical, social and psychological effects.

The aim was to explore older men’s experience and views of intimate and intrusive genitourinary investigations and specifically to develop hypotheses and theories concerning gender and sexuality issues in intimate genitourinary investigations.

**Methods:**

Written informed consent was obtained for this qualitative study. Data were collected through one-off, semi-structured interviews involving 15 men in the first year following patient’s last urological procedure. Initially, multiple themes were identified and when analysed further concepts were repeatedly present. As the urological investigations were limited to men, gender and sexuality became prominent issues in the data.

**Results:**

On analysis, the term parasexuality appeared to explain the dynamic of the situation. Parasexuality is a modified form of sexuality which is channelled and limited to maintain propriety. This was not expressed as sexuality in its overt, explicit sense, but instead a type of covert sexuality where professional boundaries are maintained but nonetheless undercurrents remain. This managed version of sexuality created a common currency by which interactions between staff and patients could take place safely.

Feeding into parasexuality were gender role stereotypes and for some of the participants this reflected their own experience, context, historical and cultural norms. Intimate contact in the form of exposure and handling of the participants' genitalia during the investigations particularly challenged the boundaries of parasexuality. In order to remain parasexual, many of the participants suppressed their sexuality. Viewing staff as professional was an additional strategy used by participants to limit any sexuality as parasexuality.

**Conclusion:**

This study has contributed towards the appeal for more studies to examine privacy perceptions of patients in genitalia-related care, however, it is by no means definitive.

Parasexuality goes some way to explain the dynamics of communication between older men and health care professionals during genitourinary investigations.

## Background

Genitourinary investigations are performed on a large proportion of middle-aged and older men and the majority undergo investigations for prostate issues. Prostate cancer is a leading cause of cancer death worldwide and the most commonly diagnosed non-skin malignancy [1]. It makes up 24% of all male cancers and causes 13% of all male cancer deaths in the UK [2]. There is a lifetime risk of 1 in 11 men getting prostate cancer before the age of 75 [3]. Old age is the strongest predictor of this disease [4] and for genitourinary investigations generally. There is a general belief that urinary symptoms are an unavoidable part of ageing [5] and for this reason men may underestimate them and their impact on sexuality.

The effects that genitourinary disease can have on men depend on the type of problem and treatment required. In addition, there are several potential iatrogenic consequences of genitourinary treatments such as impotence, gynaecomastia and urinary incontinence [3]. These disorders can have lasting devastating physical, social and psychological effects. The link between treatment for prostate cancer and reduced masculinity were investigated in a phenomenological study which found effects on impotence, libido, incontinence, body shape and energy [6]. These problems can be permanent [7], stigmatising [8], and may be underplayed and not publically aired because of their personal nature and men’s reluctance to share them [9].

The link between genitourinary investigations and men’s sexuality has been explored to a certain extent. The majority of the work explores the staff standpoint. In a study of 70 in-depth interviews a strategy for desexualising the physical examination of patients was devised which included, objectifying the patient, using a chaperone and looking professional [10]. Other issues involve how staff should manage personal care generally, including embarrassment, sexuality [11], intimacy [12,13] and even genitalia-related care [14].

Studies exploring genitalia related care from a man’s perspective are limited to their satisfaction with the service [15], and the connection between genitourinary investigations and men’s sexuality remains to be scrutinised. This was highlighted by a male urology service user that contacted the research team in 2009 to highlight his concerns regarding experiences of undergoing genitourinary investigations. His knowledge helped in the planning and design of this study. He also provided advice on recruitment, interviewing and associated issues, thus ensuring that the research was practice focused.

As a result of service user input the aim was to explore older men’s experience and views of intimate and intrusive genitourinary investigations and specifically to develop hypotheses and theories concerning gender and sexuality issues in intimate genitourinary investigations.

## Methods

Given the study aims and the lack of existing research in this area, a qualitative approach was used to underpin the study.

The study was undertaken in South-East Wales between 2009 and 2011. The genitourinary procedures in these men were diverse ranging from insertion of a catheter because of acute urinary retention to treatments such as amputation of penis following a diagnosis of cancer. Although the original focus was on investigations, participants inevitably spoke of them interchangeably with interventions and procedures.

Participants were purposively recruited into the study through adverts placed on the University website and in three regional newspapers in South-East Wales over a one year period. Potential participants who met the inclusion criteria were encouraged to contact the research team for a Research Ethics Committee (REC) approved participant information pack, which included a covering letter, participant information leaflet, consent form and a freepost envelope.

Respondents were invited to participate in the study if they were male, aged 40 years plus (as they were more likely to have experienced such medical procedures) and had recently been offered or undergone intimate, intrusive urological investigations, (e.g., urodynamics, catheterisation and digital rectal examination), which are of particular relevance to older men with prostate disease. In total, 22 men initially consented to participate in the study. However, 7 prospective participants did not respond to requests to arrange interviews and therefore did not take part in the study. Consequently, 15 men were interviewed. Participants’ age ranged from 44 to 83 years (mean 59 years). To help ensure anonymity and confidentiality limited participant demographic details are provided in Table 1.

**Table 1:** Participant demographics

**ID code**	**Urological problems/procedure(s)**
M1	Hesitancy, frequency, PSA test, rectal exam, flow etc.
M2	Frequency, digital examination
M3	Urgency, haematuria, PSA test, cystoscopy
M4	Haematuria, bladder cancer, chemotherapy, bladder reconstruction,
M5	Retention, long term catheter,
M6	Frequency, radiotherapy, hormone therapy (impotence)
M7	Foreskin problems, circumcision, self catheterisation
M8	Swollen testicles, haematuria, cystoscopy, USS
M9	Prostatectomy
M10	PSA test, cystoscopy, biopsy
M12	Blood in urine/sperm, cystoscopy
M13	Frequency, prostate cancer
M14	Haematuria, cystoscopy
M15	Circumcision, cancer, amputation of penis

### Ethical considerations

University ethics committee approval was granted in autumn 2009. As participants were recruited via advertisement only and the study did not involve the use of NHS premises or resources, NHS ethical approval was not required. Written informed consent was obtained from all participants before the first interview. Participants were assured of anonymity and confidentiality and were advised that they could stop the interviews and/or withdraw from the study at any time, without prejudice. To ensure participant anonymity and confidentiality, all participant names have been replaced by an identification code, known only to the research team.

### Data collection and analysis

The data were collected through a one-off, semi-structured interview in the first year following their last urological procedure. A pilot to the main study was undertaken on three participants to ensure that the interview schedule comprised topics that helped to inform the discussion and primarily focused on participants’ experiences of intrusive urological procedures and related issues. The data from the pilot study participants were included in this analysis.

To protect participant’s sensitivities all interviews were conducted by three experienced qualitative, male researchers (PG and two others). Most participants were interviewed in their own homes but several participants were interviewed in a private office at the University premises, at their request. Interviews lasted 30–60 minutes. All interviews were tape recorded with permission using a digital recorder and following transcription, were transferred to NVivo 10 for analysis.

The context for this research was the south Wales valleys, which has historically been a patriarchal region with the man providing for the family via manual labour, predominantly mining, with the woman staying at home child bearing and rearing. Although this picture has changed in the last generation, its influence remains. The participants were middle aged to older men covering the majority of the socio-economic spectrum having varied careers and education. Acknowledging this context is helpful in appreciating the way in which males and females, doctors and nurses were viewed in the study.

The data were collected and analysed concurrently using constant comparative analysis. Initially multiple themes were identified from the data which served the purpose of fracturing the ‘whole’. As the interviews continued to be analysed further concepts were repeatedly present. Data were further explored and themes combined. Following further analysis the concept of parasexuality was established to explain the dynamic of the situation.

The data below were gathered as a result of questions posed to elicit older men’s experience and views of intimate and intrusive genitourinary investigations. As the urological investigations were limited to men, gender and sexuality became a prominent issue in the data and so at this point it would be useful to distinguish between sex and gender. The position taken by the authors is that sex is grounded in the physical differentiation of reproductive functions whereas gender is the social/cultural differentiation of male and female functions and roles [6]. In this way sex forms a basis for gender. Sexuality is defined as ‘a central aspect of being human throughout life and encompasses sex, gender identities and roles, sexual orientation, eroticism, pleasure, intimacy and reproduction’ [16]. The term sexuality will be predominantly used in this work rather than masculinity as the expression of these qualities by men.

Selection of the data to be explored here is the responsibility of the authors. It is acknowledged that data and categories may have been omitted that had the potential to be of equal or greater significance to developing theory [17]. However, there were many references in the data to sexuality mainly manifested as parasexuality.

## Results and discussion

### Parasexuality

As data were gathered and analysed so the intersection between gender and sexuality became more prominent. However, it was not sexuality in its overt, explicit sense, but instead a type of covert sexuality where professional boundaries are maintained but nonetheless the undercurrent remains.

Eventually a central theme of parasexuality was considered as an appropriate term to express the way in which the participants recognised and asserted their own sexuality within the milieu of intimate investigations. The relationship between the participants and staff is asserted to have been parasexual with the professionalism of staff and the position of the patients stopping short of overt sexuality.

Parasexuality literally means ‘without fertilisation’ when referring to the way in which fungi reproduce. The term was adapted by Bailey in 1990 who defined it as being secondary to the main term for example, as in use of the term ‘paramedic’ [18]. Parasexuality in these data signifies ’not quite’, an attenuated form of sexuality. Bailey sought to capture the way in which women in public life were viewed in the Victorian era, specifically barmaids. The term parasexuality derives from a ‘managed’ version of sexuality where although it represented control it was acknowledged more readily and accommodated in certain sections of society particularly where women were on ‘display’ traditionally in servile or entertainment roles. In a formally patriarchal society parasexuality enabled a reworking of hegemony to allow a safe, protected situation [18].

It is argued here that a broader application of the term and the context would help to explain the responses of some of the participants whilst undergoing genitourinary investigations. For instance the ‘bar’ represents the investigations which maintained a distance between male and female staff which symbolised the ‘barmaid’; whilst the patient signified the customer.

The men were exposed and vulnerable physically and psychologically during the investigations and their sexuality potentially threatened. In order to test their sexual integrity, it is contended that they adopted a posture of parasexuality. Below are two illustrations of men exhibiting parasexual behaviour.

*‘I’ve been nursed by men and I think they’re very good but I like women (laughs)’* M13

*‘and you could think to yourself well you know there is a young lady doing this to me’* M4

Parasexuality is noted as ‘almost’ or a modified form of sexuality [18] being carefully channelled and limited to maintain propriety. This managed version of sexuality created a common currency by which interactions could take place safely within that context.

Parasexuality is described as mediating across the public and private divide [18]. In this case the body parts were normally private but the nature of the investigations drew them into the public domain as this example shows.

*‘But as I said, I wondered did she have to be there when I was sort of so vulnerable? Well I was just lying there, you know, on show’* M14

In a study examining sexuality, the body and nursing it was an expectation that their care/treatment was not defined in a sexual way [19]. The example below illustrated that patients also felt this way.

‘*These doctors and nurses they’ve seen it all. Yes, they have you know’* M8

Moderating the sexual to parasexual was evident in the data. Parasexuality allows ’everything but sex’ [18]. The genitourinary investigations with their ‘dirty image’ [19] act as a metaphorical ‘bar’ to ensure that parasexuality does not go beyond acceptable boundaries.

A major distinction between parasexuality and sexuality is distance, which works to contain its expression [18]*.* With M5 below the term ’playing around’ with his genitals by a woman denotes hetero-sexuality and comes close to the edge of acceptable parasexuality.

*‘I would have felt more uncomfortable, a bloke playing around with it than a woman, you know? It is a man thing you know? A man would rather a woman do that than a bloke I would imagine, yes’* M5

In this study, and specifically the example above, physical distance was compromised but there were many examples of symbolic distance being preserved through language, procedural etiquette, for example wearing gloves and the professional position of health care professionals.

Many of the interactions in the data had sexual connotations. The sexuality was attenuated in these circumstances to become parasexuality by the participants focussing on their illness or the immediate investigation as with M8 above. Milligan [20] explains that a situation is interpreted by those present as having (or not having) sexual connotations. Therefore it becomes the responsibility of those involved to maintain a professional stance. Although data on suppressing sexuality showed the struggle that some participants had to ensure that their investigational encounter remained professional.

*‘I always remember this, she rubs gel in it, she is scanning me (laughs) and I am thinking, this is nice’* M5

Sexual scripts may be a useful framework to explain the application of parasexuality [21]. Sexual scripts are said to occur on three levels. First there is a cultural scenario, for example genitourinary investigations. Superimposed on that are interpersonal scripts. Interpersonal scripting of these data enabled the individuals to remain actors in a passive, parasexual role rather than to become part ‘scriptwriter’ in a more sexual, active role. The third level of intra-psychic scripting remains private to those engaged in it and involves fantasy. M12 (in the ‘Suppressing Sexuality section below) explains that during an examination by the doctor (cultural script) he made an effort to remain in a passive, parasexual role (interpersonal scripting) but concurrently had thoughts regarding homosexuality (intrapsychic scripting).

### Gender role stereotypes

Feeding into parasexuality were gender role stereotypes and the expectations that the participants had of the professionals caring for them. Some of the participants gender-stereotyped staff which reflected their own experience, context, historical and cultural norms. Throughout history nurses have been predominantly female. Predating Florence Nightingale, those tending the sick, wounded and dying have been recorded and portrayed as women. Gender role stereotyping was loosely connected to the socio-economic group of the participants with those having had manual employment more likely to stereotype. It was unsurprising then that the participants tended to expect doctors to be male and nurses female. Garmarnikow [22] articulated the nurse, doctor, patient triad as resembling the power relationships between the father, mother, child relationships within a family. Within this model each role had specific boundaries.

*‘I feel nursing is a woman’s job. I’ve been nursed by men and I think they’re very good but I like women (laughs). As long as they’re good at their job that’s all I’m interested in’* M13

*‘You know because a woman is a nurse to you. You think from your childhood a nurse is a woman’* M4

Perhaps because of these social norms, female nurses tended to be the preference. Using the Garmarnikow model, being nursed or treated by a woman presents potential challenges when the male patient (the child) potentially becomes vulnerable, exposed and weakened as a result of their investigations [19].

*‘I’m not seeing a doctor now, I goes to (the local cancer centre) but all I see is the urology nurse but I can’t ask this man, you know he’s a lovely man don’t get me wrong but I don’t think he’s qualified enough for me to explain the symptoms to him you know so then again you can’t ask for the doctor because he’s too busy so (laughs) a bit strange you know’* M6

Despite the above example relating to a male nurse, continuing with the family analogy [22], the nurse’s role reflects that of the woman within the household as the carer and nurturer. This oppression of nursing, as a predominantly female profession within the triad, can be seen above where the participants hinted at nurses’ sexuality and underestimated their expertise (even though he was male). Interestingly, there was no evidence of the role of mother/nurturer extending to female doctors, confirming the deep-rooted stereotypes associating doctors with being male [10].

As seen above, participants distinguished between the expertise of nurses and doctors and this seemed to override the gender divide. The male nurse, however experienced, did not seem to have the same credibility or authority as the doctor. Some participants acknowledged that gender influenced their decision to ask for information about their condition. When asked why he wouldn’t ask the (male) nurse about his problems of gynaecomastia and impotence, he added.

*‘Well, I’d be so embarrassed about it and I mean it seems unmanly you know the man is supposed to be butch and we don’t talk about things like that’* M6

M6 not only subscribes to the notion that a doctor would be able to provide more information than the nurse, but also that being a man precluded him from taking on the role of the dependent child.

### Intimate contact

Exposure and handling of the participants' genitalia during the investigations particularly challenged the boundaries of parasexuality. In this study the majority of participants spoke about the importance of privacy, but they recognised that it would be compromised during investigations. Intimate contact manifested itself differently throughout the study depending upon the stage of investigations and included contact with urine plus exposure and handling of participants’ genitalia by staff. After being catheterised by a nurse this participant was then asked to catheterise himself. He was asked how he felt.

*‘Terrible! I mean I am not normally embarrassed, but I felt then as if they were there to you know like she sort of put me on show to have a laugh. I don’t know, I felt that because they were sort of looking at one another and smirking and I felt you know. It was an embarrassing thing to have to do anyway even if it had just been her. To have these student nurses there and I thought they had sort of come around for a laugh’* M7

This intimate contact plus the added humiliation added to his embarrassment. In a dated, German study patients found care by students more embarrassing than by trained nurses and offered the rationale that students have a closer connection to their social identity than their professional one Bauer (1994) cited in [11]. This reflects the comment by M7 as it was conceivable that he was unable to view the students as fully professional. Bauer 1994 cited in [11] also suggests that students have not yet learned to manage their embarrassment which could explain the looking and smirking that M7 describes as adding to his distress.

Another participant spoke about his emotional discomfort when three women prepared him for the ‘camera’ by the consultant.

*‘I’ve been examined by women before and it’s never been a problem but I just did feel, it was such a personal intrusion, you know, what they had to do. They had to apply anaesthetic and it was so personal, it just made me shudder a bit’* M11

As with M7 the professionals were handling his penis which is the most intimate of acts occurring between a male and female. This type of contact rates as highly intimate on Carnaby and Cambridge’s [23] classification in their study of personal and intimate care. Intimate contact breaches privacy [24] but the two participants above expected it to be maintained during intimate contact.

M11 went on to describe that he could not explain why he felt the way he did.

*‘normally I’m pretty good in situations like that but I think maybe if there had been two (instead of three) and maybe if they had been a little bit older’* M11

Age influenced intimate contact with several participants who stated that they would have preferred an older member of staff to perform the procedure. They may have been because of their greater experience. Although it may also have reflected Fultz and Herzog’s [25] claim that gender differences decrease over adulthood expressed as the ‘androgyny of later life’. Pateman and Johnson [9] established that men found intimate care less embarrassing from an older woman. One participant in this study claimed that he would have been more sensitive to the investigations had he been younger perhaps strengthening the hypothesis of androgyny of later life [25].

Intimate contact more often than not included exposure of the genitals and quite often exposure went beyond what was necessary.

*‘They carried out the procedure but I’m lying down horizontal and the doctor asked me to pull my trousers and my underpants down to my toes and I’m just lying there if you like open to the world um and I did think at that time you know, ‘is this the only way that this can be done’?*’ M14

Goffman's [26] exposition on embarrassment helps to explain the discomfort of many participants especially as he asks who is likely to suffer the embarrassment (the patient) and who causes it (the professional)? The male nurses in one study [27] sought strategies to protect themselves from accusations of inappropriate touch by setting a formal tone, shaking the patient's hand prior to care, wearing a uniform and modifying techniques to minimise the need for intimate touch. There is little in the literature explaining useful strategies for patients in this situation.

Sexuality helps to define an individual and for many it is at the core of their personhood. For men masculinity is said to centre predominantly on the penis. Lawler [19] explains that the role of the body and sexual organs in the majority of social situations is invisible and its exposure is controlled. However, in these investigations participants’ bodies were exposed to a range of strangers and private functions such as urination became public. It is likely that this exposure and vulnerability was of significance to these men because of the impact on their perception of their manhood.

Interestingly, the proposition put forward regarding parasexuality is that patients represent the customer and staff, the barmaid. However, when examining the dynamics of intimate contact the position of barmaid and customer seemed to be reversed with the patients feeling on show and vulnerable as a barmaid might.

### Suppressing sexuality

Parasexuality relies on all participants in the scenario knowing the limits of the phenomenon. In order to remain parasexual, many of the participants suppressed their sexuality. A latent sexuality triggered many of the examples given regarding investigations. Despite conceding this the participants were at pains to stress that it remained implicit and was never expressed to staff. This could be said to be an example of a reflexive action where the participants, at risk of breaching the boundaries of parasexuality, tried to limit it by suppressing their sexuality.

The participant below happened to be impotent.

*‘I could see a clock up there and I always remember this she rubs the gel in it, she is scanning me (laughs) and I am thinking this is nice and they got another woman washing me so then she said to me have you any objection to my colleague because she is training, I said no none whatsoever. So I am looking at the clock and I thought and so I come out laughing to the wife and she says what have you been up to now? I said you don’t have to go down for a massage I said I have had it on the National Health here! (laughs). And I thought no, no problem at all because I think, I think they are so, it is the way they, they are so well trained’* M5

In this example, M5 rapidly retrieves a situation which appears to be overtly sexual by endorsing the professionalism of the staff (see below).

Oliffe’s [7] qualitative research into masculinity in post-prostatectomy men discusses the profound link between the penis, sexuality and masculine status which if extrapolated to parasexuality acknowledges it as having a fallo-centric component. The quote below sums up some of the other comments in revealing the struggle against embarrassment and erection in suppressing sexuality in the presence of female members of staff.

*‘I think I was more embarrassed because I wasn’t impotent then (laughs) and you could think to yourself ‘well you know there is a young lady doing this to me’, nothing like that occurred obviously but that is in the back of your mind’* M4

This participant was not alone in fearing getting an erection as the investigations involved touch of their sexual organs (see below). Intimate touch is noted as a risky act which is open to misinterpretation [13] and the difficulties of this forced intimacy is acknowledged in the literature.

This participant made health his priority, but acknowledges that in other circumstances viewing a nurse in a sexual way would be worthy of consideration.

*‘I think if you are a well man who hasn’t got any symptoms then yes looking at a nurse in a tight nurses uniform is a different matter but when you are ill it is not the first thing on your mind’* M3

For some participants the link between their investigations and sexuality inhibited their ability to express themselves or gain information. This was evident with participant M6 in the Gender Role Stereotype section above. He was unable to discuss his problems of gynaecomastia and impotence with the staff ostensibly because of gender and associating an ascribed role to doctors but also possibly because of the close association of his problems with his sexuality. The example below highlights how suppressing sexuality when communicating with staff can also suppress communication.

*‘Before the operation Professor (Consultant’s name) said you will be impotent after the operation and I knew what it meant. I read, looked it up, everything. He explained that to me, but I thought afterwards I could have talked a little bit about it to somebody, but I have never been. I sort of tried to bring it up a couple of times but it just doesn’t. I haven’t taken that step to ask out right so…’* M4

Suppressing their sexuality to lessen embarrassment was a common theme. Unfortunately for M4 it limited his chances of receiving accurate advice or treatment for his condition.

The ability to get an erection unites male gender with sexuality and masculinity. According to Lawler [19], impotence becomes a highly stigmatising and stressful experience for some men as it can dismantle their masculine image. Some participants spoke about their impotence but it is likely that some did not know whether this would occur following their investigations and treatment which could have caused them uncertainty and anxiety. At this point it is worth making a distinction between libido and impotence in that even if a man loses the ability to gain an erection, he may still retain his libido, or desire for sex.

Suppressing sexuality was prominently related to heterosexuality, but this participant amongst others revealed his fear of being the subject of a homosexual fantasy.

‘Um, again a certain level of kind of embarrassment and self consciousness; and thinking things like God I don’t want to get a f***ing erection like (laughs). It was terrible like, you know what I mean; and funnily enough the overriding thought of that (coughs) and that some sense of common sense in me was like come on this is just something medical, let’s get it done with. But I had to kind of consciously think not to be embarrassed and not to feel awkward about the situation, whereas when I saw the guy in the hospital who was a male for the prostate thing – there was a certain amount of that but not so much you know. Having said that, this sounds very awful, but I had thoughts going through my head at the time, thinking I wonder if this guy’s into this? When he does this, he’s quite comfortable with the process – it may be something that he’s into (laughs)’ M12

Zang et al’s [14] literature review found that some men were perceived to be homophobic as they disliked being touched by a male nurse. This supports the persistent label of male nurses as being homosexual. Interestingly, in this study homophobic views seem to have been perpetuated with several comments echoing the fear of homosexuality which were not restricted to nurses.

Garaminkow’s [22] model of the health care triad as family does not take account of potential homosexuality in any of the members. None of the participants disclosed that they were homosexual and no homosexual encounters were described in the data. Nonetheless, it cannot be assumed to be absent. The invisibility of potential homosexuality in patients has been discussed in the literature [28,29]. Interestingly, neither publication cited discusses this issue in relation to health care staff.

Little has been previously written about patients’ need to suppress their sexuality during intimate investigations. However, there has been discussion on the suppression of sexuality by some professionals when physically examining patients [10]. The authors cite three desexualising strategies used by staff from their research: meeting the patient clothed beforehand, engaging in non-sexual joking and using medical (not colloquial) terms [10].

Not all participants needed to suppress their sexuality and for some this topic did not arise. However, for those that did they considered staff as professionals and they were able to make a distinction between social and professional interactions during the investigations.

### Professionalising staff

Viewing staff as professional was a strategy used by participants to limit any sexuality as parasexuality. This was used in addition to the tactic of suppressing their sexuality.

*‘I also think it (professionalism) negates gender to some extent umm you know it is all very well that Barbara Windsor, the sexy nurse thing on the carry on films and so on, but in reality I think it the last thing that is passing through your mind when all of that is going on’* M3

There were multiple examples of praise for the professionalism of staff and within that there was rationalisation of the need for professionalism to ‘trump’ any sexual connotations.

*‘They were doing their job and doing it to me and that was it. I could understand why some people would find it embarrassing if either a male or a female was doing it, I can understand that because there was some people in the hospital who wouldn’t even let students watch when they were having things done to them’* M4

Professionalising staff reduced sexuality to the more innocuous parasexuality in order to make encounters during investigations manageable.

‘No I think they are so well trained and it is how they do it. I had no problem, it wasn’t embarrassing whatsoever. How she done she threw a cloth over you, over the other part and done that and she told me it is a lot better now’ M5

There is some literature from the staff perspective regarding professionalism. For example, Milligan [20] using Foucault’s premise stated that by maintaining a professional gaze, the deep psychosocial consequences of genitourinary investigations may be disregarded. The need for patients to professionalise staff has been little explored and this finding adds to the body of knowledge in this area.

### Limitations of the study

This study was limited to investigating male genitourinary investigations. Despite a wealth of literature exploring the feelings of women undergoing gynaecological examinations, particularly related to childbirth, there is little to authenticate their experiences when undergoing urinary tract procedures specifically. This is an area ripe for exploration with the opportunity to compare findings with this study.

In retrospect it would have been fitting to have offered the participants a choice of female or male interviewer in keeping with the sensitivities of the topic [6]. Offering further interviews to the participants at a later date may have gleaned a deeper level of data as a phenomenological study of men post-prostatectomy found they were initially satisfied with their care and treatment [9] and only later did they give a more realistic picture of their experiences.

### Rigour

External validity, or generalisability of the existence of parasexuality across populations is not possible following this research [30]. However, it is argued that there is adequate evidence of internal validity, or the degree to which the data support the evolving theory. Achieving reliability in qualitative research is more challenging and is often termed credibility in qualitative research. The researchers adhered to a set of principles for ensuring rigour or credibility [31]. The reader is left to decide whether this has been achieved (Table 2).

**Table 2:** **Adapted from Chiovitti and Piran** [32]

**Standards of rigour**	**Suggested methods of research practice**	**Methods to ensure rigour in this study**
Credibility	1. Let the participants guide the inquiry process.	Constant comparative analysis and data collected over one year.
	2. Check the theoretical construction generated against the participants’ actual words in the theory.	
	3. Use participants’ actual words in theory.	Verbatim quotes used in the findings section.
	4. Articulate the researcher’s personal views and insights about the phenomenon explored by means of:	
	a. Post-comment interview sheets used as a tool.	Reflexive journal used.
	b. A personal journal	Literature was used in the background and discussion.
	c. Monitoring how the literature was used	
Auditability	5. Specify the criteria built into the researcher’s thinking.	An outline of method used.
	6. Specify how and why participants in the study were selected.	Outlined in section on participants.
Fittingness	7. Delineate the scope of the research in terms of the sample, setting and level of the theory generated.	Delineated in the abstract and main body of article.
	8. Describe how the literature relates to each category which emerged in the theory.	Using the categories as headings, literature was drawn upon in the discussion.

## Conclusion

This study has contributed towards the plea for more studies to examine privacy perceptions of patients in genitalia-related care [14], however, it is by no means definitive.

The phenomenon of parasexuality was adapted to describe the dynamic occurring between staff and patients during genito-urinary investigations. This was a managed version of sexuality which created a common currency by which interactions between staff and patients could take place safely.

As a modified, attenuated form of sexuality, parasexuality ensured that despite gender role stereotypes professional boundaries were maintained. During intimate contact patients used strategies such as suppressing their own sexuality and viewing staff as professionals to ensure that parasexuality did not deteriorate into sexuality.

Bailey warns against reifying the term parasexuality [18]. In keeping with this, there is a need to investigate the concept of parasexuality in more detail than this study has allowed such as the juxtaposition of staff as barmaid and patient as customer in the understanding of this phenomenon.

Other considerations in future work would be how parasexuality develops over the trajectory of an illness with intimate episodes and how this is learned by patients and indeed staff. It may be that the intervention (in this case genito-urinary investigations) becomes the key to attenuation of sexuality to parasexuality. Nevertheless other avenues need to be explored before this can be confirmed.

## Competing interests

The authors declare that they have no competing interests.

## Authors’ contributions

AL drafted the majority of the manuscript. CS conceived of the study, participated in its design and coordination and edited the manuscript. PG participated in study design and coordination, performed data collection, drafted some of the manuscript and commented on it. All authors read and approved the final manuscript.

## Authors’ information

All authors having a nursing background. They all have a specific research interest in user experiences in health care, particularly in relation to urinary tract.

AL is Principal Lecturer, CS is Reader and PG is Post-Graduate Research Training Co-ordinator, all are based at the University of South Wales, UK.
